# Supplementary material for: Increasing occurrence of cold and warm extremes during the recent global warming slowdown
Source: Nat Commun. 2018 Apr 30;9:1724. doi: 10.1038/s41467-018-04040-y (PMC5928063; doi:10.1038/s41467-018-04040-y)
Supplement: Supplementary file 1 — Supplementary Information [file 41467_2018_4040_MOESM1_ESM.pdf]

SUPPLEMENTARY INFORMATION

Increasing occurrence of cold and warm extremes during the recent global warming slowdown

Johnson et al.

Supplementary Figures

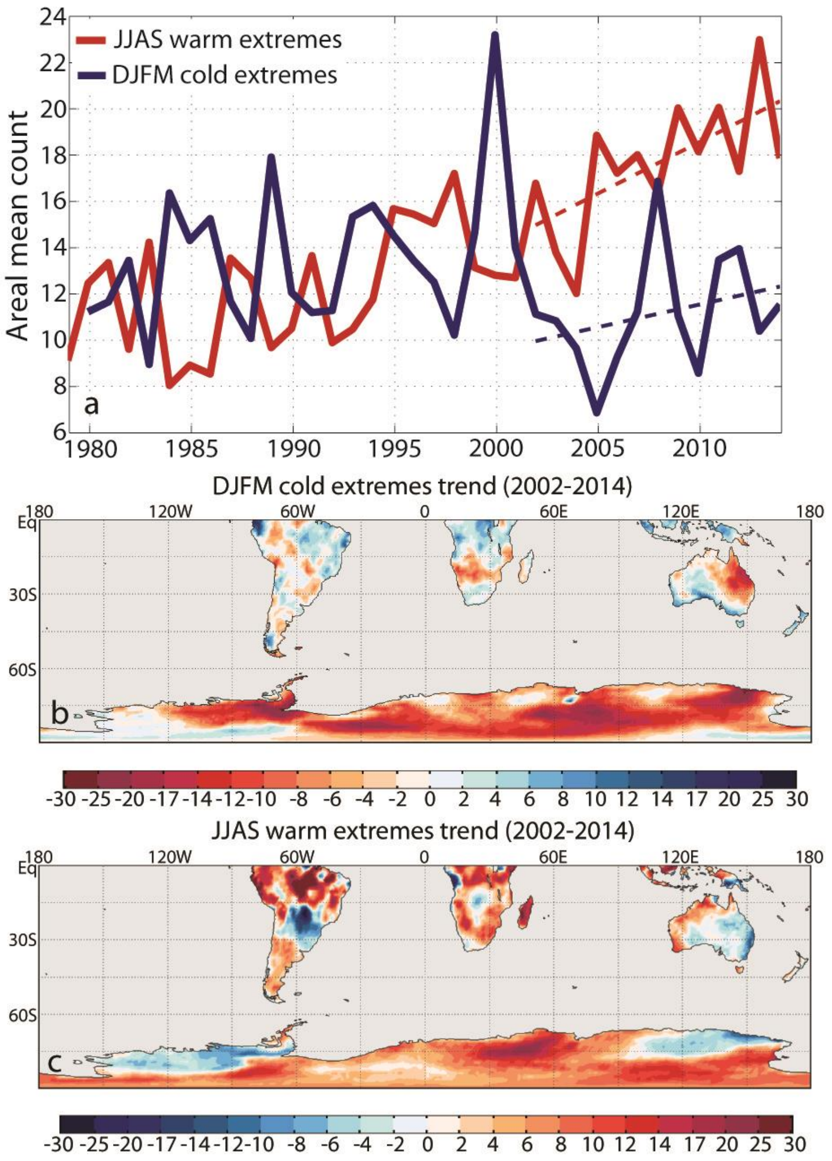

Supplementary Figure 1 | Linear trends of Southern Hemisphere summertime cold and wintertime warm extreme occurrence during the recent global warming hiatus. (a) Summertime (December-March, DJFM) cold (blue) and wintertime (June – September, JJAS) warm (red) extreme temperature occurrences (d season^{-1}) over Southern Hemisphere land from 1979-2014. Temperature extremes are defined by the 10th and 90th percentiles of the local ERA-Interim temperature anomaly distributions (see Methods). Dashed lines indicate the least squares linear trend during the hiatus period of 2002-2014 (4.5 d [10yr]$^{-1}$ for JJAS TX90d and 2.0 d [10yr]$^{-1}$ for DJFM TX10d). (b) Linear trends of summertime cold extreme temperature occurrence and (c) wintertime warm extreme temperature occurrence (d [10yr]$^{-1}$) during 2002-2014 at each Southern Hemisphere land grid point.

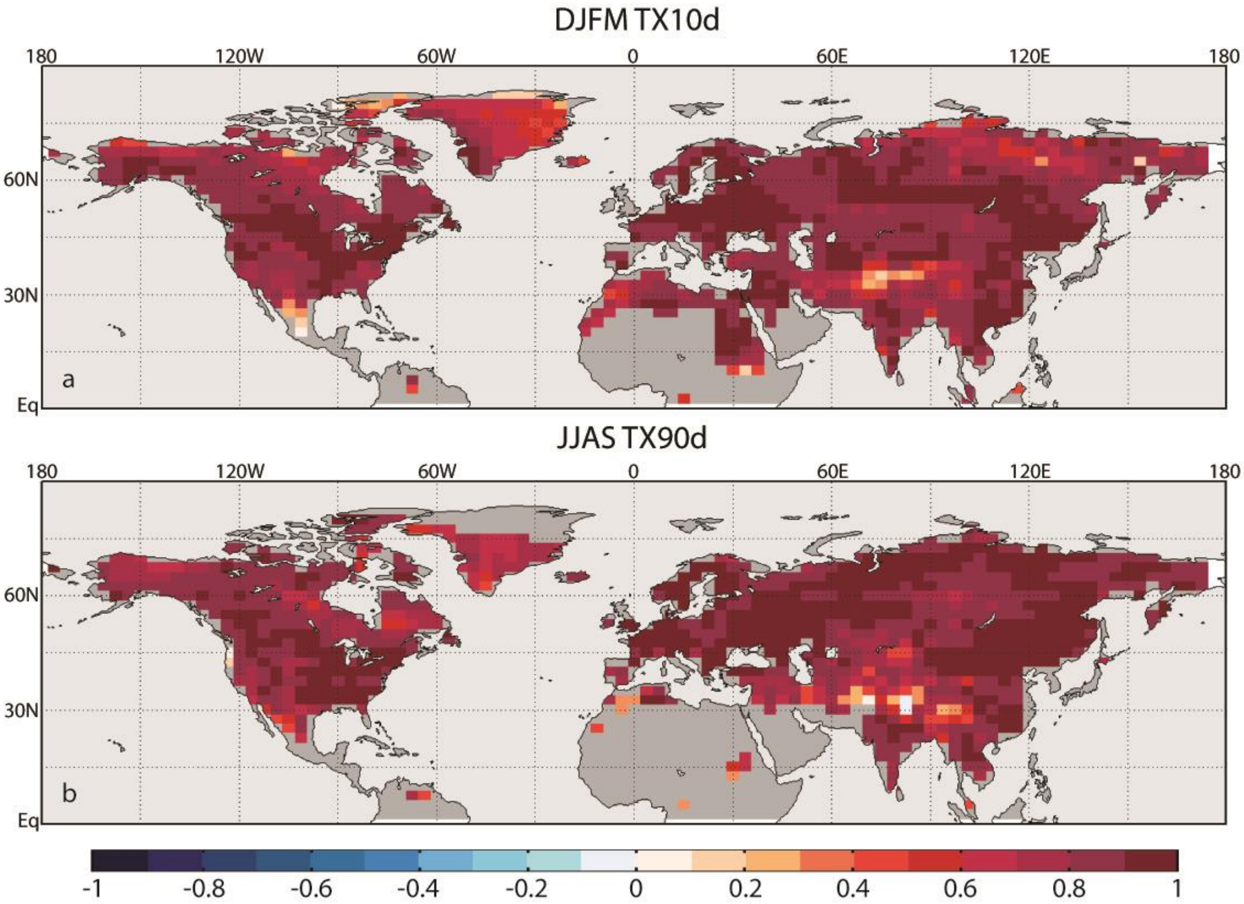

Supplementary Figure 2 | Correlations between HadEX2 and ERA-Interim cold and warm extreme occurrences. Correlations between HadEX2 and ERA-Interim (a) DJFM TX10d and (b) JJAS TX90d during the 1979-2010 period of overlap. Darker gray indicates land regions not included in the reconstructed dataset owing to insufficient HadEX2 coverage.

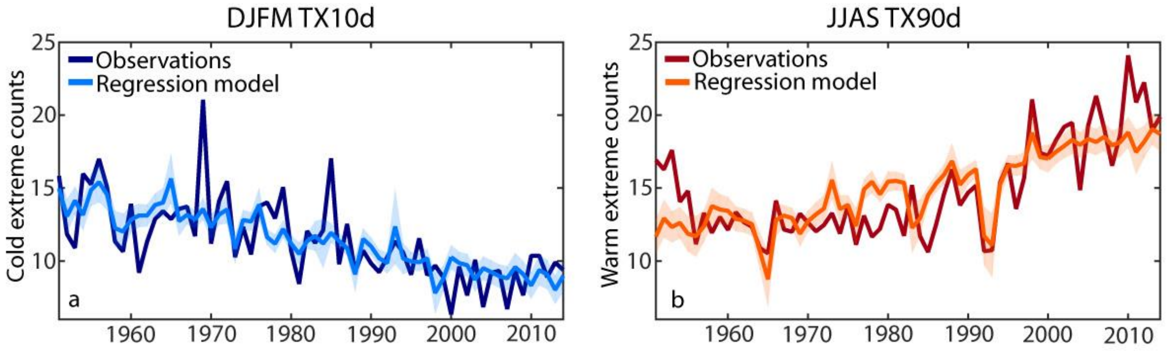

Supplementary Figure 3 | Multiple linear regressions of Northern Hemisphere wintertime cold and summertime warm extremes. (a) Time series of actual (dark blue) and regressed (light blue) DJFM TX10d from 1951-2014. (b) As in (a) but for actual (red) and regressed (orange) JJAS TX90d. The regressions are based on the three predictors described in the Methods section. Light blue and orange shading represents the 95% confidence interval of the fitted values. For TX10d the correlation between the actual and regressed values is 0.72 with all three predictors and 0.56 if time is excluded as a regressor. For TX90d the corresponding correlations are 0.76 and 0.50, respectively.

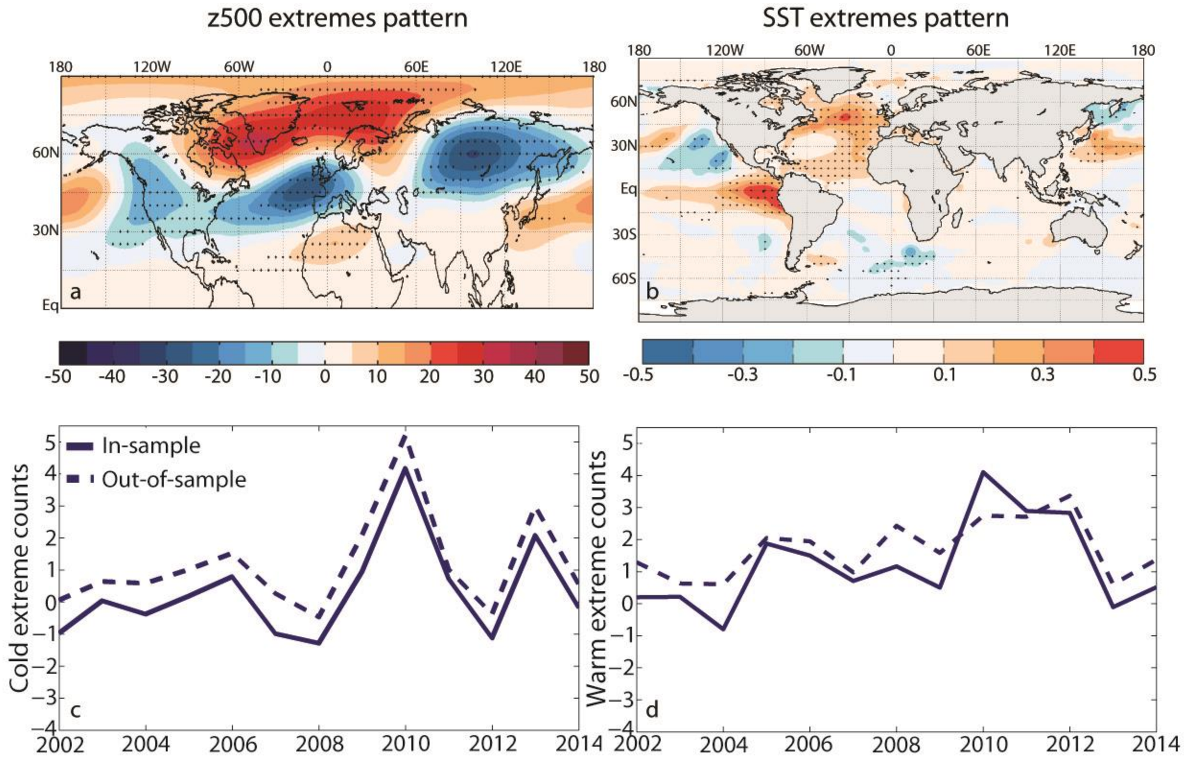

Supplementary Figure 4 | The z500 and SST extremes patterns from data excluding the hiatus period. The (a) z500 and (b) SST extremes patterns, shown as partial regression maps (units of m and °C, respectively) and calculated in the same way as in Fig. 3 but from 1951-2001 data only. The in-sample contribution of the (c) z500 extremes index to DJFM TX10d and the (d) SST extremes index to JJAS TX90d for the 2002-2014 hiatus period are shown as solid blue lines (same values as in Fig. 2). The predicted contributions of the (c) z500 and (d) SST extremes patterns to DJFM TX10d and JJAS TX90d, respectively, during the 2002-2014 hiatus period based on regression parameters determined from 1951-2001 data are shown as dashed blue lines (see Methods for additional details).

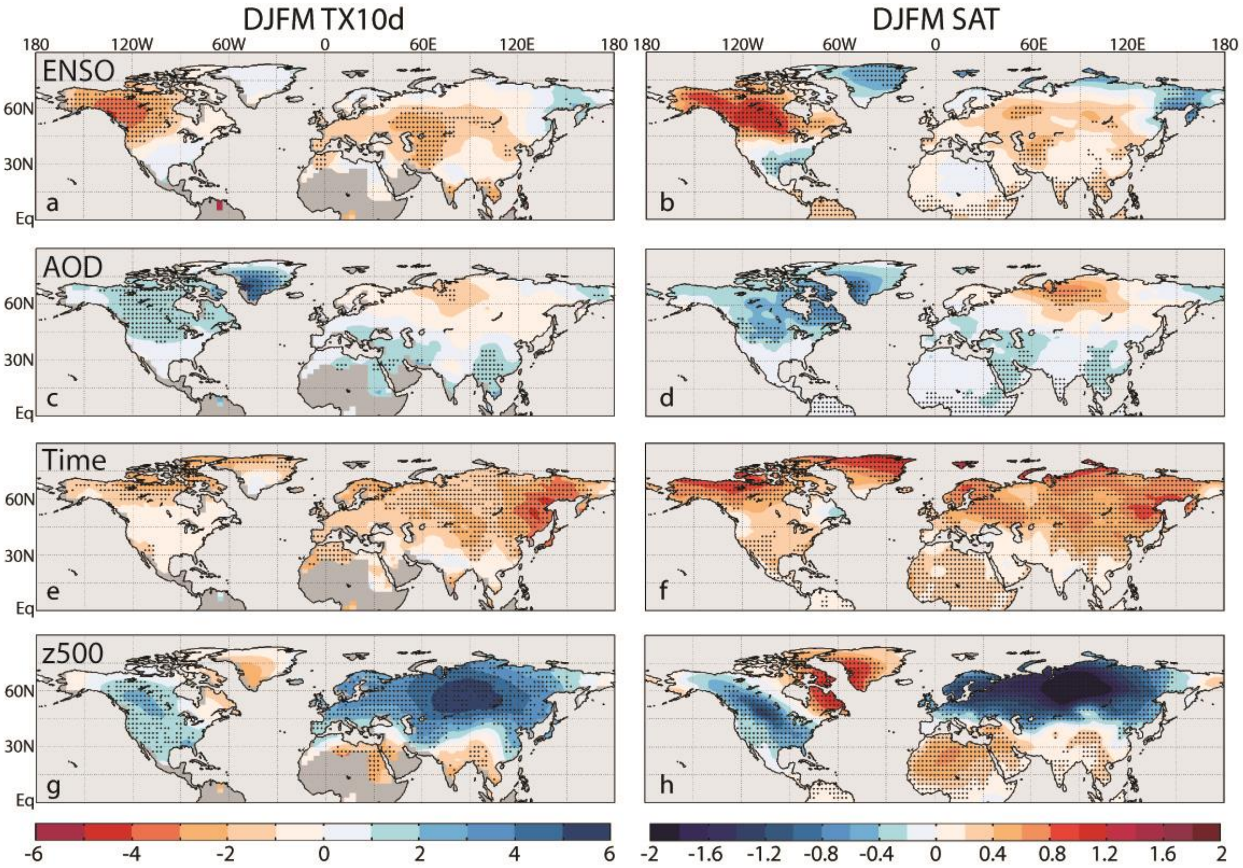

Supplementary Figure 5 | Partial regression maps of wintertime extreme cold occurrences and mean surface temperature on the predictors of hemispheric cold extreme occurrences. Partial regression maps of December – March (left) TX10d (d) and (right) mean surface air temperature (K) on (a,b) ENSO, (c,d) volcanic AOD, (e,f) time trend, and (g,h) the z500 extremes index. All predictor indices are identical to those used in the regressions on hemispheric TX10d (see Methods).

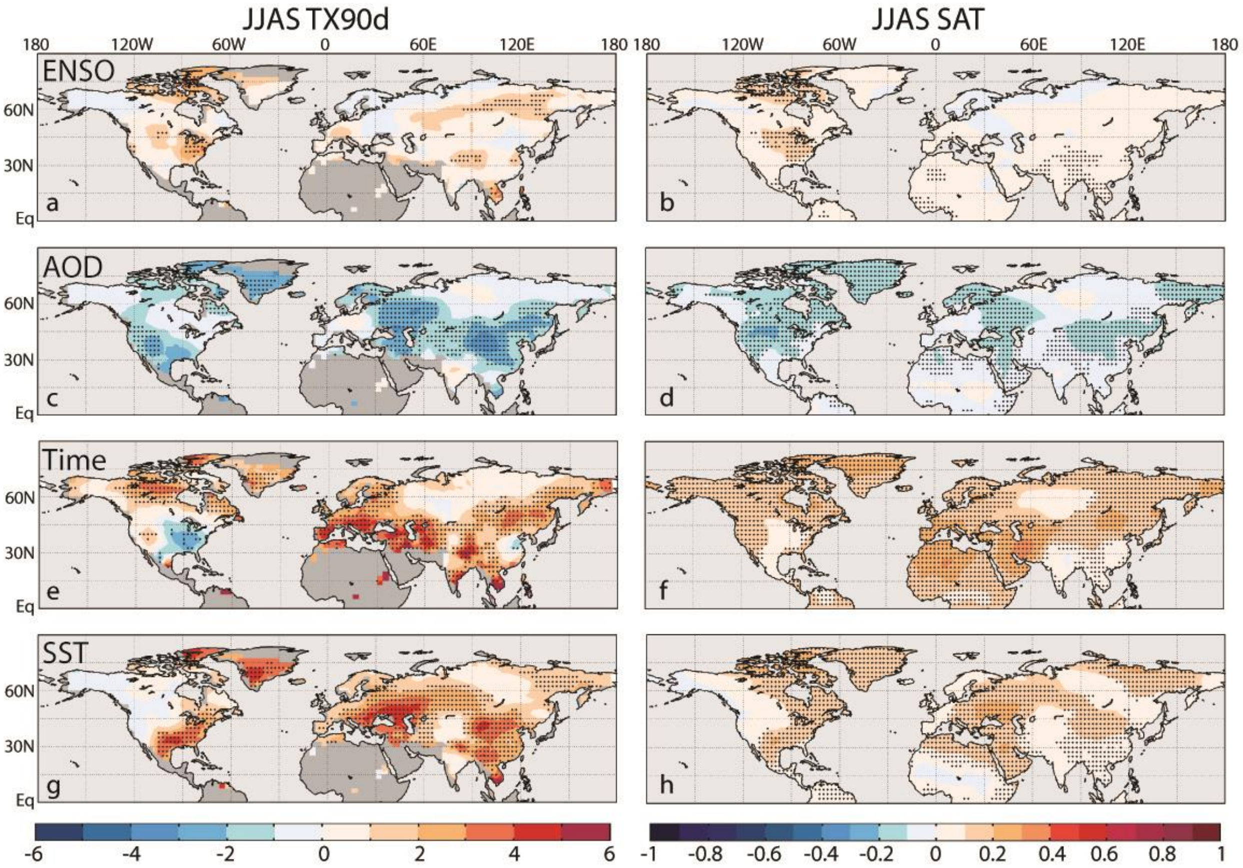

Supplementary Figure 6 | Partial regression maps of summertime extreme warm occurrences and mean surface temperature on the predictors of hemispheric warm extreme occurrences. Partial regression maps of June – September (left) TX90d (d) and (right) mean surface air temperature (K) on (a,b) ENSO, (c,d) volcanic AOD, (e,f) time trend, and (g,h) the SST extremes index. All predictor indices are identical to those used in the regressions on hemispheric TX90d (see Methods).

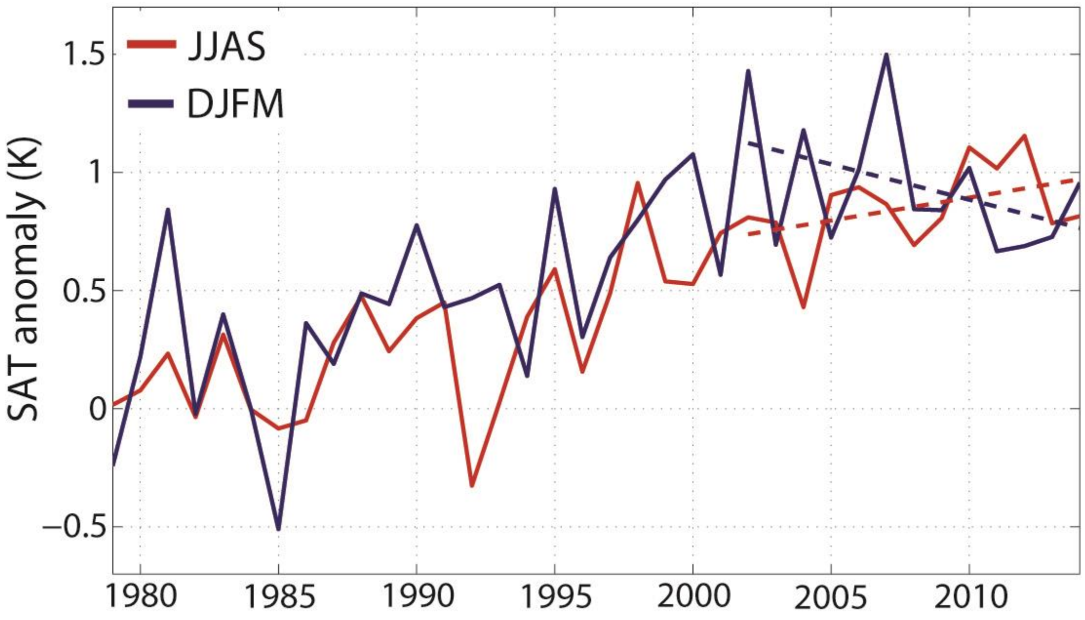

Supplementary Figure 7 | Hemispheric land seasonal mean temperature time series. Time series of area-averaged December – March (blue) and June – September (red) NH land surface air temperature anomalies (K) since 1979. The dashed blue and red lines indicate the 2002-2014 linear trend lines.

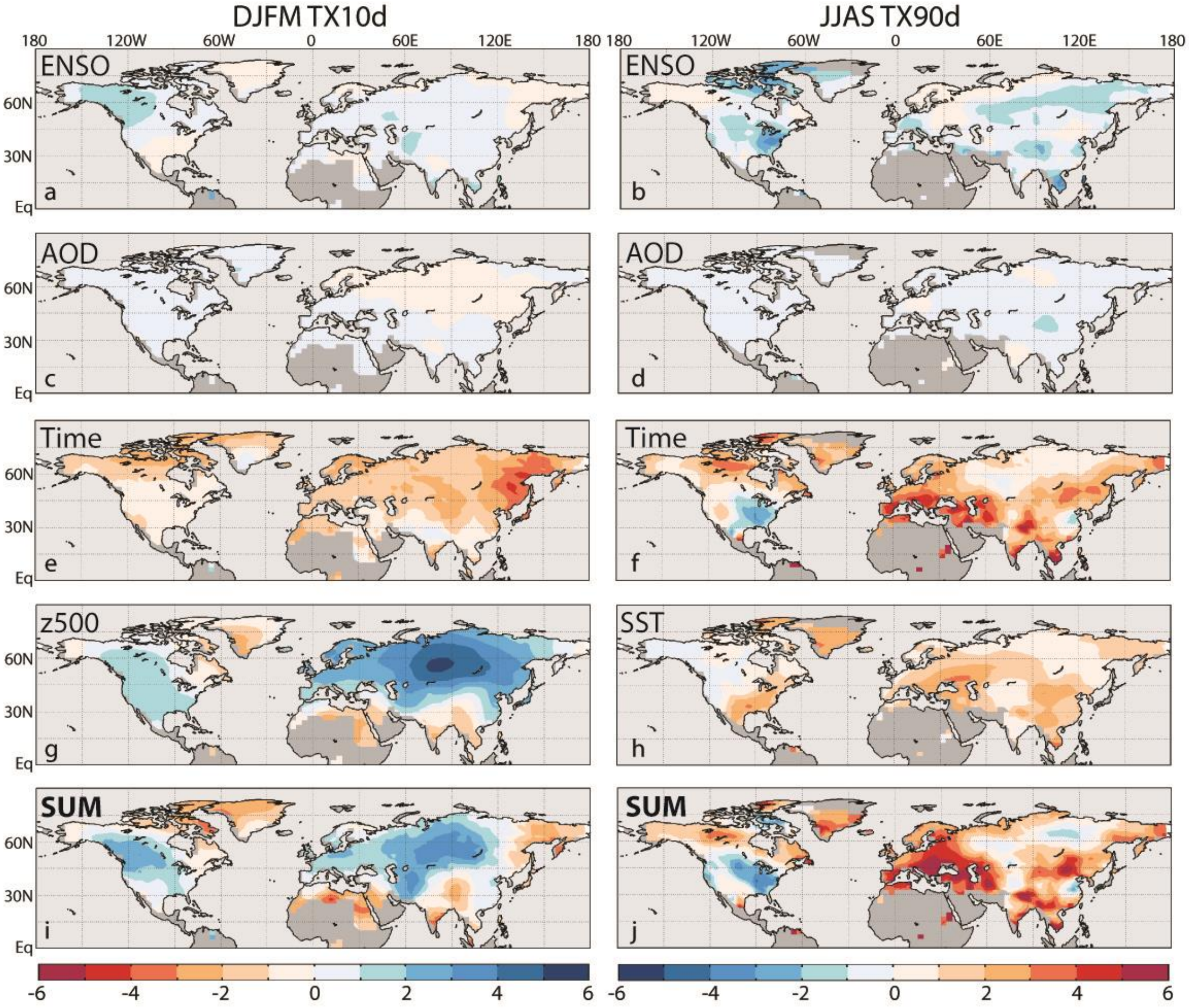

Supplementary Figure 8 | Contributions of individual predictors to the cold and warm extreme occurrence trends during the hiatus period. (a-h) Contribution of individual predictors to the (left) December – March TX10d and (right) June – September TX90d 2002 – 2014 linear trends (d $[10\ yr]^{-1}$) as obtained by multiplying the partial regression maps in Supplementary Figs. 5 and 6 by the residual predictor linear trends (see Supplementary Note 2). (i-j) The sum of the four components for (i) TX10d and (j) TX90d.

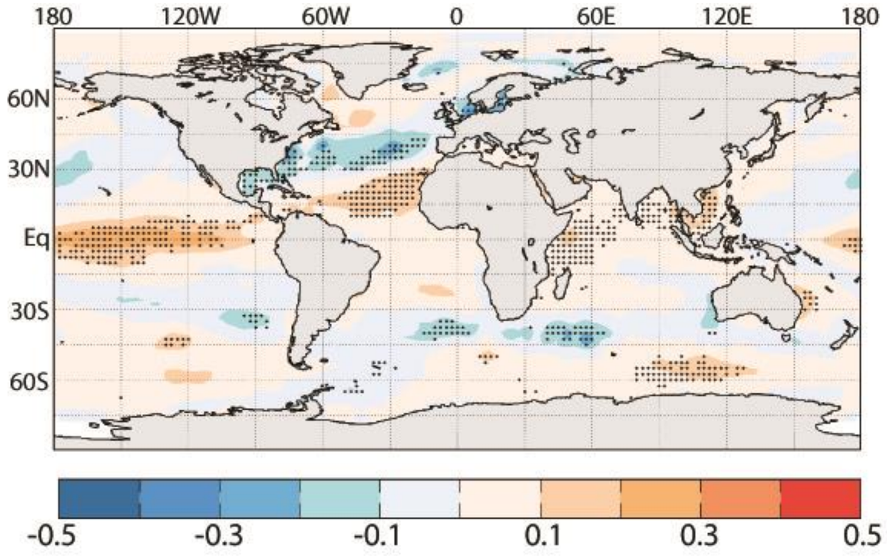

Supplementary Figure 9 | Relationship between the z500 extremes index and SSTs. Partial regressions of December – March SST anomalies (°C) on the December – March z500 extremes index. Stippling indicates statistically significant regression coefficients at the 5% significance level.

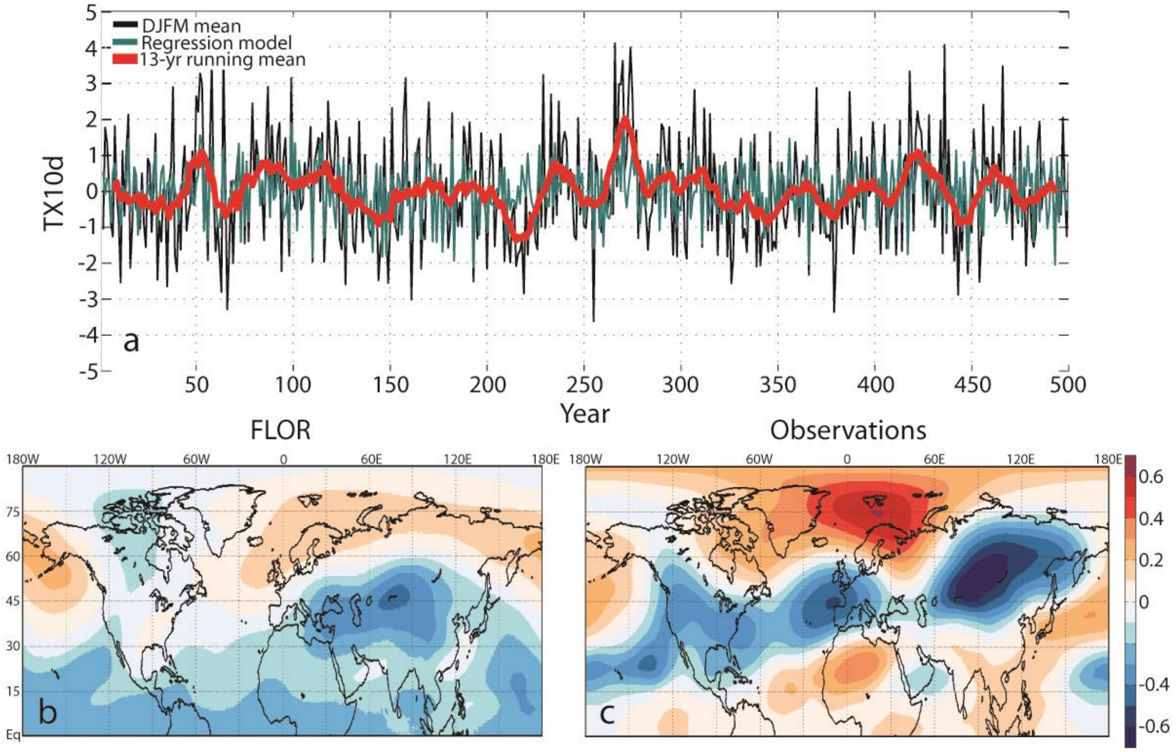

Supplementary Figure 10 | Variations in wintertime extreme temperatures in a 500-yr control simulation. (a) Time series of the December – March TX10d anomalies (d, black) with the 13-yr running mean (red) for the FLOR simulation with constant radiative forcing. The green line is the regression of TX10d with two predictors, the Niño 3.4 SST index and the z500 extremes index (see Supplementary Note 5 for details). (b) The grid point correlations between DJFM z500 and TX10d after linearly removing the influence of ENSO in the 500-yr FLOR simulation. (c) The grid point correlations between DJFM z500 and TX10d after linearly removing the influence of ENSO, time, and volcanic AOD in observations.

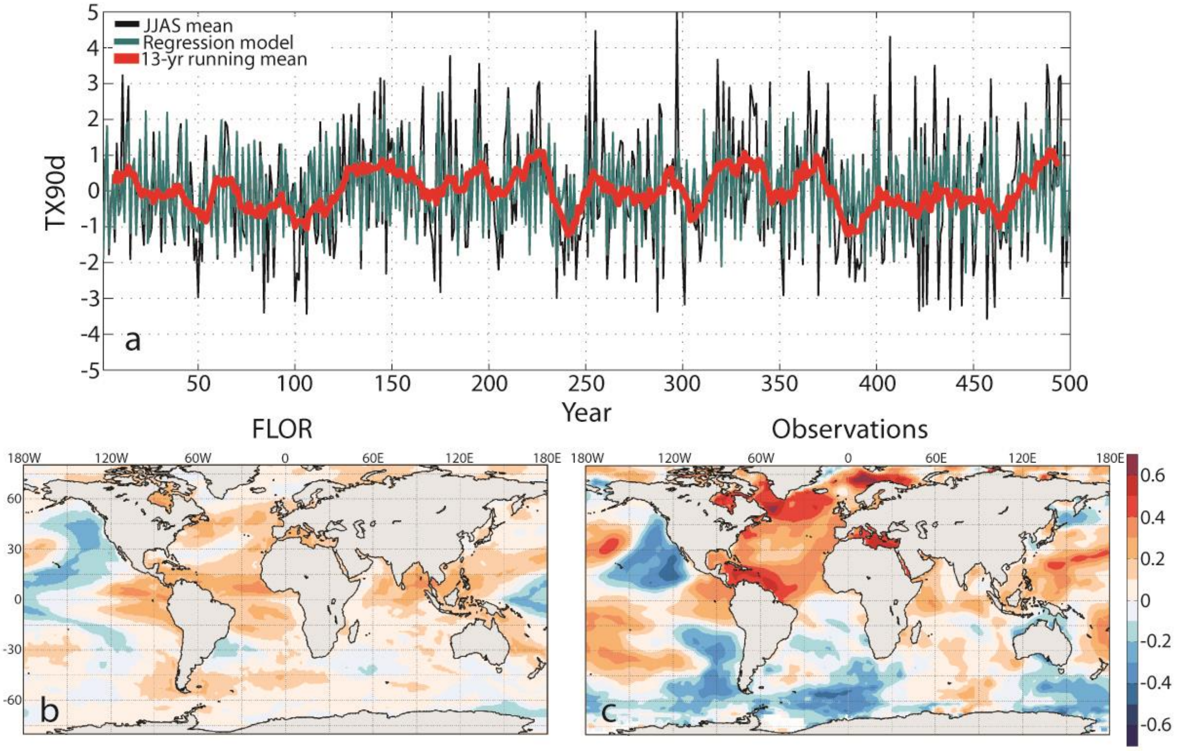

Supplementary Figure 11 | Variations in summertime extreme temperatures in a 500-yr control simulation. (a) Time series of the June – September TX90d anomalies (d, black) with the 13-yr running mean (red) for the FLOR simulation with constant radiative forcing. The green line is the regression of TX90d with two predictors, the Niño 3.4 SST index and the SST extremes index (see Supplementary Note 5 for details). (b) The grid point correlations between JJAS SSTs and TX90d after linearly removing the influence of ENSO in the 500-yr FLOR simulation. (c) The grid point correlations between MJJ SSTs and TX90d after linearly removing the influence of ENSO, time, and volcanic AOD in observations.

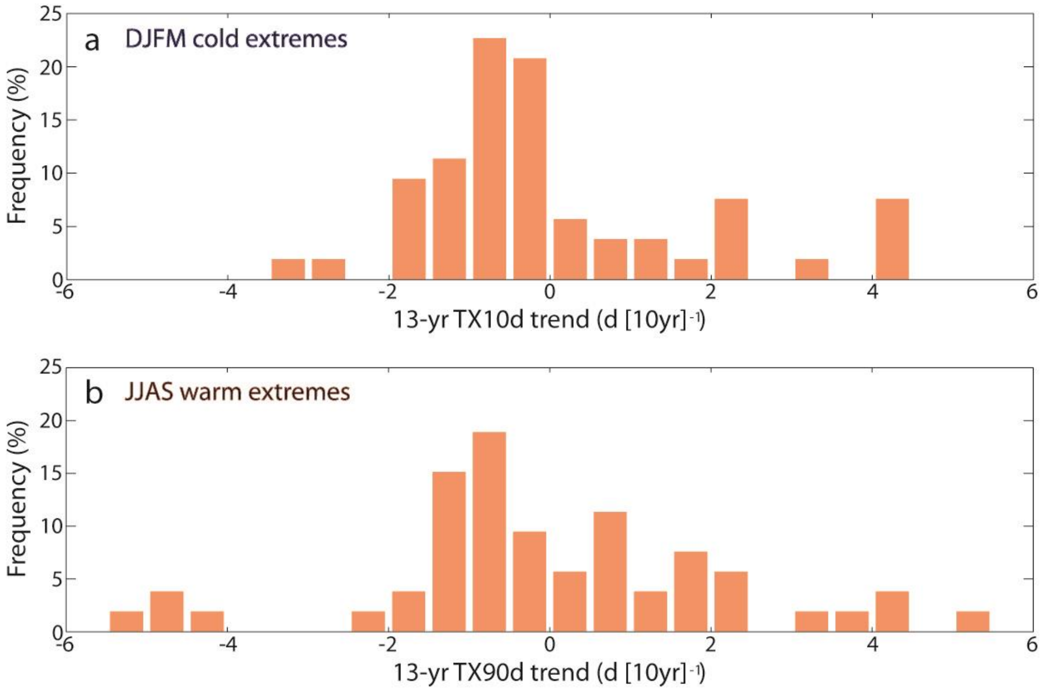

Supplementary Figure 12 | Distribution of observed 13-yr trends in wintertime cold and warm extreme occurrences. Histogram of the (a) 13-yr DJFM TX10d and (b) 13-yr JJAS TX90d trends in the linearly detrended 1951-2014 observed datasets.

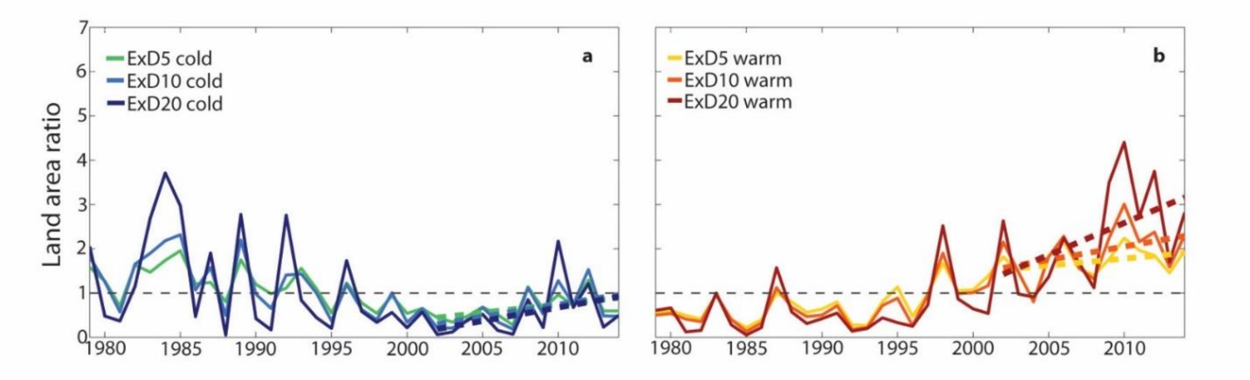

Supplementary Figure 13 | Time series of NH land area affected by exceedances of cold and hot extremes. Time series of the ratio of NH land area affected by the exceedances of 5, 10, and 20 (a) December – March extreme cold days and (b) June – September extreme warm days relative to the 1979-2010 average. The dashed black line at a land area ratio of 1 corresponds with the 1979-2010 average. The dashed colored lines indicate the 2002-2014 linear trend lines; the 2002-2014 trends for cold (warm) extreme exceedances are 0.037 (0.031), 0.055 (0.060), and 0.061 (0.144) $[10\ yrs]^{-1}$ for ExD5, ExD10, and ExD20, respectively. See Supplementary Note 6 for details.

Supplementary Note 1. Extending HadEX2 with ERA-Interim reanalysis

As discussed in the Methods section, we face the challenge that the HadEX2 dataset features more than 60 years of data, but the record ends in 2010, which excludes the last four years of the hiatus period studied here. To extend the HadEX2, we reconstruct TX10d and TX90d counts through 2014 through linear regression on ERA-Interim values following equation [1] in the Methods section. This reconstructed dataset only includes regions for which HadEX2 has at least 90% temporal coverage. To demonstrate that the correspondence between HadEX2 and ERA-Interim data is strong enough to perform this extrapolation, we show in Supplementary Fig. 2 the gridded correlations between HadEX2 and ERA-Interim values during the period of overlap (1979-2010).

The correlations generally are greater than 0.8 across most of the hemisphere, confirming that the ERA-Interim reanalysis is a suitable proxy for the HadEX2 dataset for this purpose. The most notable exception is the region around the Himalayas, where the correlations are rather low. Nevertheless, the small area with poor agreement should not impact any results significantly, particularly since the extrapolation is only for four of 64 years.

Supplementary Note 2. Partial regression maps for individual predictors

In order to elaborate on (1) the connection between extreme temperature occurrence and seasonal mean temperature and (2) the role of each predictor on the spatial pattern of temperature extreme trends during the hiatus period, we present partial regression maps of extreme temperature occurrence and seasonal mean temperature for each predictor of NH land mean temperature extremes. We use seasonal mean land surface temperature for the period 1951-2014 from the Berkeley Earth Surface Temperature project[1] which are monthly land surface temperature data on a 1° latitude-longitude grid. Partial regression maps are calculated in the following way: the partial regression map for predictor *i* is obtained by first linearly regressing out the influence of all predictors excluding *i* and then calculating the regression of the residual values at each grid point on residual predictor *i*. The predictors are identical to those used for the regressions of area mean temperature extreme occurrences (see Methods). Supplementary Figures 4 and 5 provide the partial regression maps of seasonal extreme temperature occurrences and seasonal mean temperature for boreal winter (Supplementary Fig. 5) and summer (Supplementary Fig. 6).

Overall, we see a rather close correspondence between the changes in extreme temperature occurrence and in the seasonal mean anomalies, which is consistent with expectations that changes in extreme temperature occurrence closely follow changes in seasonal mean temperature[2,3]. The time series of hemispheric, seasonal mean land temperature since 1979 (Supplementary Fig. 7) closely mirrors the TX10d and TX90d time series of Fig. 1 (r = -0.86 for DJFM and r = 0.96 for JJAS). Regionally, there are some exceptions, such as the negative linear trend of JJAS TX90d over the eastern United States despite positive seasonal mean temperature trends; this region is known to have experienced negative trends in daily maximum temperature

that are much lower than those of the mean temperature and a reduction in extreme temperature occurrence[4]. The regional temperature variations associated with ENSO and volcanic AOD are consistent with previous studies (e.g., refs. 5 and 6).

Next, we seek quantitative evidence that z500 and SST extremes patterns, in combination with the long-term trend, can capture the overall observed trends in TX10d and TX90d, respectively, during the hiatus period of 2002-2014. For this analysis, we multiply the partial regression maps in Supplementary Figs. 5 and 6 by the 2002-2014 linear trend of the corresponding predictor to determine an estimate of that predictor contribution to the 2002-2014 linear trends of TX10d and TX90d. The results of these calculations are shown in Supplementary Fig. 8. Consistent with Fig. 2, both ENSO and the AOD make generally minor contributions to the trend of continental extreme temperature occurrence. Both the linear trend and z500 and SST extremes patterns make substantial contributions to the hiatus period trends. The sum of the four contributions to the hiatus period trends (bottom of Supplementary Fig. 8) agree well with the trend pattern from ERA-Interim data (Fig. 1), albeit with some regional differences. For example, the regression results do not explain the 2002-2014 negative trends in summertime warm extreme occurrences over northwest Europe and central Asia. Nevertheless, the overall results of this analysis support that the z500 and SST extremes patterns are dominant contributors to the spatial variations in extreme temperature occurrence trend variations during the hiatus period.

Supplementary Note 3. Relationships with canonical indices

As indicated in the partial regression plots of Fig. 3, the z500 extremes pattern is related to the NAO and AO patterns, whereas the SST extremes pattern is related to the AMO pattern. This observation leads to the following questions: (1) how distinct are the z500 and SST extremes indices from these canonical indices? (2) How well would the canonical indices explain the variation in cold and warm extreme occurrences relative to the z500 and SST extremes indices? The answers to these questions would determine whether it truly is worth defining these new patterns and indices or if the existing indices provide a sufficient existing framework for understanding hemispheric extreme temperature occurrence.

To address these questions for the z500 extremes pattern, we first downloaded the NAO and AO indices from the National Oceanic and Atmospheric Administration (NOAA) Climate Prediction Center (CPC) website. The NAO pattern is determined from a rotated principal component analysis of 500 hPa geopotential height poleward of 20°N, whereas the AO pattern is determined as the leading empirical orthogonal function of 1000-hPa geopotential height poleward of 20°N. We then calculated the correlation coefficients between the z500 extremes index and December – March NAO and AO indices for the 1951-2014 period. The correlation between the z500 extremes index and the NAO (AO) index is -0.65 (-0.68), confirming the expectation that the z500 extremes pattern is strongly related to these canonical patterns but also indicating that a substantial fraction of the z500 extremes pattern variability cannot be explained by either the NAO or AO index.

We next address the degree to which the NAO or AO index can explain the wintertime variation in TX10d, which also addresses whether the NAO or AO index may be a suitable replacement for the z500 extremes index. After linearly removing the influence of the other

predictors (time trend, ENSO, and volcanic AOD) from the NH DJFM TX10d time series, the z500 extremes index explains 56% of the residual TX10d variance. The NAO and AO indices, however, only explain 17% of the residual TX10d variance. This finding indicates that the z500 extremes pattern, although strongly related to the NAO and AO, is much more closely tied to wintertime cold extreme occurrences than either the NAO or AO. Therefore, the z500 extremes pattern may represent a part of the NAO or AO continuum[7,8] most closely related to the wintertime cold extreme occurrences over land. Although the NAO index has been invoked to explain recent cold European winters during the hiatus period[9], neither the NAO nor AO index can capture the recent increase in hemispheric cold extreme occurrences as completely as the z500 extremes index.

As discussed in the main text and in the preceding section, the patterns of extreme temperature occurrence are closely tied to the patterns of seasonal mean temperature. We therefore also address the degree to which the DJFM NH continental mean temperatures also related to the three pattern indices. As mentioned in the main text, the correlation between the z500 extremes index and the linearly detrended NH land mean surface temperature is -0.54, confirming a strong relationship. The corresponding correlations for the NAO and AO index are 0.19 and 0.26, respectively. Again, the z500 extremes index is much more closely related to seasonal mean continental temperatures than either the NAO or AO index despite similar regression patterns with surface air temperature (not shown). This finding provides a justification for considering the z500 extremes pattern as an NAO- or AO-like circulation pattern closely tied to warm Arctic and cold continental conditions.

We follow a similar procedure to compare the SST extremes index with the AMO index. We define the AMO as the area averaged SST from 0-60°N, 0-80°W minus SST from 60°S-

60°N (ref. 10). We also considered an alternative version, defining the AMO as the SST averaged from 25-60°N, 7-75°W minus the regression on global mean temperature[11]. The relationships between the SST extremes index and this alternative AMO index are consistent with those of the definition of ref. 10, and so we focus only on the first definition. The correlation between the MJJ SST extremes index and the AMO index is 0.66. Therefore, like the arguments raised above, the SST extremes index is significantly related to the AMO but there is substantial variability of the SST extremes pattern that cannot be explained by the AMO. The Pacific component of the pattern, which, as mentioned in the main text, resembles the "Pacific Extreme Pattern" (ref. 12), appears to be an important component that is unique from the AMO despite the prominence of Atlantic SST anomalies in the SST extremes pattern.

Next, we determine that the SST extremes pattern explains 75% of the residual JJAS TX90d after the removal of the linear influence of the other predictors. The AMO index explains only 35% of the residual TX90d variance. Like the arguments raised above about the z500 extremes pattern, the SST extremes pattern explains the variation in hemispheric temperature extreme occurrences much better than the AMO despite the strong relationship between the two patterns. Similarly, the SST extremes index is more closely related to seasonal mean hemispheric land temperature. The correlation between the SST extremes index and the linearly detrended JJAS NH land temperature is 0.68, whereas the correlation for AMO is only 0.20. These findings indicate the utility of maintaining the distinction between the AMO and SST extremes pattern.

Supplementary Note 4. Possible mechanisms of the z500 extremes pattern

Although Fig. 6 confirms an association between autumn BK Sea ice anomalies and a wintertime z500 pattern tied to cold temperature extremes, a relationship that is consistent with many recent studies[13-20], establishing a possible causal link and the associated physical mechanisms remains a challenge. An emerging physical argument for a BK Sea ice/atmospheric circulation link has focused on a possible stratospheric/tropospheric coupling pathway[14,17,21,22], although tropospheric pathways relating to changes in baroclinicity and synoptic storm activity may also play a role[19,20,23]. Essentially, a reduction in BK Sea ice loss may alter autumn and wintertime planetary waves to increase stratospheric wave driving, which results in a weakened stratospheric vortex and a circulation response that propagates downward and projects onto the negative phase of the AO once the response manifests in the troposphere. If the stratospheric/tropospheric coupling mechanism is valid, there is some debate about whether the planetary wave modification relates directly to the sea ice anomalies or indirectly via changes in snow cover[14,24].

Despite the observational and modeling support for a link between Arctic sea ice loss and midlatitude weather, doubts about this connection remain[25]. As shown in Fig. 3c of the main text, the z500 extremes pattern exhibits strong interannual and interdecadal variability throughout the entire period, not just the recent period of rapid sea ice loss. This evidence of strong internal climate variability makes it challenging for a sea ice-forced signal to stand out from the noise of natural variability. In addition, some Atmospheric Model Intercomparison Project (AMIP)-type climate model studies, where observed SIC are imposed as the atmospheric forcing, fail to reveal a strong impact of the observed sea ice decline on the large-scale

atmospheric circulation[26-30]. These findings have led to the conclusion that the recent wintertime continental cooling is largely a manifestation of internal atmospheric variability.

Although this contrarian perspective raises many valid points, we should exercise restraint when confidently attributing recent trends to internal atmospheric variability based on AMIP simulations. The observed cooling over Eurasia during the past 15-25 years tends to lie well in the left tail or even outside of the envelope of the AMIP ensemble[26-28], which means that observed Eurasian cooling would be a very rare or even an unprecedented event in this framework. Given how unusual such an event would be if it were explained solely by internal atmospheric variability, we also must consider the possibility that the models are underestimating the atmospheric response to sea ice forcing or underestimating the amplitude of internal climate variability (see Supplementary Note 5). Given the many gaps in our understanding and the diversity of climate model responses to sea ice anomalies, this topic likely will remain an active area of research.

As mentioned in the main text, an alternative hypothesis is that the La Niña-like SST pattern in the tropical Pacific during the hiatus period may have forced a Rossby wave response that projects onto the negative phase of the NAO, resulting in more frequent cold air outbreaks over Eurasia[9]. Given this hypothesis, we also examine the partial regression coefficients of the December – March ERSSTv3b SST anomalies on the z500 extremes index (Supplementary Fig. 9), where we again have linearly removed the TX10d predictors from the gridded SST prior to the correlation calculations. These calculations reveal that the z500 extremes pattern is associated with weak though statistically significant positive SST anomalies in the eastern equatorial Pacific. This relationship is consistent with the warm ENSO/negative NAO relationship examined in many studies[31]. The partial regression pattern in Supplementary Fig. 9,

however, clearly contrasts the negative SST anomalies in the eastern equatorial Pacific that dominated the hiatus period. Therefore, we find no evidence that tropical Pacific SSTs played a role in the predominance of the z500 extremes pattern during the hiatus period.

Supplementary Note 5. Analysis of a 500-yr climate model simulation

Here we examine further the variability of wintertime cold and summertime warm extreme occurrences in the 500-yr control simulation of the GFDL FLOR model. Despite fixed radiative forcings, the DJFM TX10d and JJAS TX90d time series exhibit substantial decadal to multidecadal variability (Supplementary Figs. 10a and 11a). We now address whether these internally generated variations of TX10d and TX90d are associated with similar large-scale climate variations as in the analysis of observational data.

Following the approach we use for the observational data, we attempt to develop regression models that can explain a large fraction of the FLOR TX10d and TX90d variability. Because radiative forcings are held constant in the simulation, we consider only two potential linearly independent predictors, ENSO and either z500 for TX10d or ENSO-removed SSTs for TX90d. We are particularly interested in determining whether a WACC-like z500 extremes pattern and an AMO-like SST extremes pattern emerge in the 500-yr FLOR simulation as they do in the observations. To determine the ENSO part of the regression, we follow the same procedure as with the observational analysis by finding the lag with the maximum correlation strength between the DJFM TX10d or JJAS TX90d and the seasonal Niño 3.4 SST index. For TX10d, this maximum occurs at a lag of -2 months (October – December, OND) and a correlation of -0.24. This correlation is similar to that of observational TX10d and the Niño 3.4

SST index (r = -0.21), although the maximum correlation amplitude in observations occurs at a lag of -8 months (AMJ, r = -0.31). For TX90d the maximum occurs at a lag of -2 months (April – June, AMJ) and a correlation of 0.53. The relationship between ENSO and JJAS TX90d is stronger and at a different lag in the FLOR model than in observations. In the observational record, the maximum correlation with the Niño 3.4 index is 0.36 at a lag of -10months (ASO), and the maximum correlation with the MEI is 0.32 at a lag of -11 months (JAS).

We next follow the PLSR procedure described in the Methods section to determine a predictor in addition to ENSO for TX10d and TX90d. For DJFM TX10d, we first linearly remove the OND Niño 3.4 index from the TX10d time series and the gridded DJFM z500 fields. We then calculate the correlation between the residual TX10d and gridded z500, and project the standardized z500 anomalies onto the correlation map. For JJAS TX90d, we follow the same procedure but with the removal of the AMJ Niño 3.4 index and for the calculation of an additional SST predictor. The optimal lag for the SST predictor turns out to be lag 0 (i.e., the simultaneous JJAS), which is only slightly different from the observational analysis, where the maximum correlation occurs at a lag of -1 month (MJJ). The results of this analysis, however, change very little if we choose a lag or zero or -1 month. The z500 (SST) projection time series become the second predictors in the linear regressions of TX10d (TX90d).

Supplementary Figures 10 and 11 present the linear regression results for the FLOR control simulation. Supplementary Figure 10b (11b) illustrates the correlation between the residual DJFM TX10d (JJAS TX90d) time series and the residual DJFM z500 (JJAS SSTs) in the 500-yr simulation. The observational counterparts are shown in Supplementary Figs. 10c and 11c, although the observational correlation maps are based on time series that also have removed the influence of volcanic AOD anomalies, and the SST correlation map in

Supplementary Fig. 11c (the correlation form of the SST extremes pattern in Fig. 3b) is based on MJJ rather than JJAS SSTs.

For wintertime cold extremes, the FLOR model does reproduce many of the features of the z500 extremes pattern (Supplementary Fig. 10b), including a high-over-low z500 dipole over Eurasia. However, the correlations in FLOR generally are weaker, and not all of the features are reproduced, such as the extent of negative correlations over North America and southwest Europe. Consequently, the two-predictor regression model (Supplementary Fig. S10a) does not capture as strong a relationship between the actual and regressed TX10d (r = 0.51) as in observations (r = 0.80 after linearly detrending both time series in Fig 2a). This analysis of the 500-yr control simulation allows us to conclude that the z500 extremes pattern arises as a leading pattern of internal climate variability that explains a substantial portion of the seasonal occurrence of wintertime extremes, but some of the spatial features and the strength of the connection to hemispheric extreme temperature occurrences are not reproduced in the simulation. As discussed in the main text, a possible reason for some of the discrepancies may relate to the underestimate of NAO-related internal climate variability in the model.

Next, we focus on the companion analysis for JJAS TX90d and the emergence of a naturally occurring SST extremes pattern in the 500-yr FLOR simulation (Supplementary Fig. 11b). The peak North Atlantic and North Pacific SST correlations based on observations (Supplementary Fig. 11c) are stronger than those of FLOR. However, the correlation patterns are remarkably similar across the entire Northern Hemisphere, which indicates that the SST extremes pattern is an important source of internal variability of TX90d in the 500-yr FLOR simulation.

The two-predictor regression model of JJAS TX90d (Supplementary Fig. 11a) performs better than that of DJFM TX10d, with a correlation between the actual and regressed TX90d of 0.69. This value is somewhat lower than that of the actual and regressed TX90d in observations (Fig. 2b; r =0.91 after linearly detrending both time series). As in the TX10d analysis, the observational analysis, however, includes the influence of another important predictor, volcanic AOD. In addition, the observational regression fits more predictors to a shorter record, which would inflate the in-sample correlations more than in the FLOR analysis. These two factors indicate the true difference in regression performance is not as large as may appear at first glance. In any case, these results, which are consistent with the observational analysis, indicate that ENSO and the SST extremes patterns are dominant sources of summertime extreme temperature variability that are purely internal to the climate system. These findings suggest that a substantial portion of the multidecadal TX90d variability associated with the SST extremes pattern may be a result of natural, unforced climate variability, although we still cannot exclude an important role for anthropogenic aerosol forcing.

Overall, we see that Northern Hemisphere, wintertime and summertime temperature extreme occurrences exhibit pronounced interannual and interdecadal variability owing to internal climate variability. However, we may question if the amplitude of decadal, internal climate variability in the FLOR simulation is realistic. To address this question, we show histograms of 13-yr trends of DJFM TX10d and JJAS TX90d from the observational, linearly detrended 1951-2014 time series (Supplementary Fig. 12). Overall, the distributions of observed 13-yr trends are wider than their FLOR counterparts (Fig. 4), although only the observed wintertime TX10d distribution is significantly different from the corresponding FLOR distribution at the 5% level on the basis of a two-sample Kolmogorov-Smirnov test. The

underestimate of wintertime TX10d variability in the FLOR simulation likely relates, at least in part, to an underestimate of the variability of the z500 extremes pattern (Fig. 5). This finding raises two possibilities: (1) FLOR, and by extension of ref. 30 to many other climate models, systematically underestimate the low-frequency internal variability of the z500 extremes pattern, or (2) the manifestation of the z500 extremes pattern as a dominant control of hemispheric cold extreme occurrences in observations may not entirely owe to internal atmospheric variability (see Supplementary Note 4).

Supplementary Note 6. Recent changes in 'extreme extremes'

In this study we have focused on a moderate definition of extremes by examining the occurrences of temperature anomalies in the top and bottom 10% of the distribution, which have closely followed the seasonal mean temperature anomalies (Supplementary Note 2). A more selective definition of extremes, however, may yield contrasting behaviour in recent trends. Indeed, the most extreme hot extremes ('extreme extremes') have risen faster than more moderate hot extremes during the recent global warming slowdown[32]. In this section we examine if this claim holds for both summertime hot and wintertime cold extremes over the Northern Hemisphere land regions. We reproduce the analysis of ref. 32 but with a few key differences: (1) we consider both hot and cold extremes, (2) we partition the year into boreal summer (June – September) and boreal winter (December – March), and (3) we focus only on the Northern Hemisphere.

Following the methodology described in ref. 32, we calculated the extreme warm and cold day exceedances (ExD) for various thresholds with ERA-Interim reanalysis temperature

data. The measure ExD is based on the land area with TX10d or TX90d exceeding various occurrence thresholds. First, we calculated the reference number of extreme warm and cold occurrences (ExDref) at each grid point, which is the average JJAS TX90d or DJFM TX10d for the 1979-2010 reference period, and then determined warm and cold occurrence anomalies at each grid with respect to ExDref. We next calculated the land area of extreme occurrence anomalies exceeding 5 (ExD5), 10 (ExD10), and 20 (ExD20) days. These thresholds overall are lower than those considered by ref. 32 (ExD10, ExD30, and ExD50) because we are counting totals over four-month seasons instead of a calendar year. Finally, we calculate the ratio between ExD land area in a given year and the average ExD land area in the reference period (1979-2010).

Supplementary Figure 13 shows the land area ratio time series for DJFM cold extremes (Supplementary Fig. 13a) and JJAS hot extremes (Supplementary Fig. 13b). Consistent with ref. 32 for annual data, the more extreme measures of hot extremes have increased faster than the more moderate hot extremes during the hiatus period. In particular, the 2002-2014 linear trend of ExD20 (0.144 yr^{-1}) is over four times as large as that of ExD5 (0.031 yr^{-1}). For cold extreme occurrences, however, the contrast is not as stark. The linear trends in ExD5 and ExD10 for cold extremes during the hiatus period are comparable to those of hot extremes. The trend of ExD20 for cold extremes (0.061 yr^{-1}), however, is much lower than that of hot extremes and is similar to that of ExD10. Therefore, the tendency for the most extreme temperature occurrences to increase more rapidly than more moderate extremes[32] holds for summertime hot extremes in the Northern Hemisphere but not for wintertime cold extremes.

These findings suggest that the strong correspondence between seasonal mean temperature and extreme temperature occurrences during the hiatus period found in this study

may not hold as well for more extreme measures of summertime hot extremes but is likely to be robust for wintertime cold extremes. The reason for the amplified response of the hottest extremes requires more study, but soil moisture-temperature feedbacks are one plausible culprit[33], although there is some indication that this mechanism for accelerated warming of hot extremes in climate models may not hold in observations, at least in some regions[34]. Overall, the degree to which extreme temperatures follow the changes in the mean of the temperature distribution may depend on the region, season, type of extreme (hot or cold), and the threshold used to define the extremes.

Supplementary References

1. Rohde, R. *et al.* Berkeley Earth Temperature averaging process. *Geoinfor. Geostat: An Overview* **1**: doi:10.4172/gigs.1000103 (2013).

2. Rhines, A. & Huybers, P. Frequent summer temperature extremes reflect changes in the mean, not variance. *Proc. Nat. Acad. Sci.* **110**, E546 (2013).

3. Argüeso, D., Di Luca, A., Perkins-Kirkpatrick S. E. & Evans, J. P. Seasonal mean temperature changes control future heat waves. *Geophys Res. Lett.* **43**, 7653-7660 (2016).

4. Portmann, R. W., Solomon, S. & Hegerl, G. C. Spatial and seasonal patterns in climate change, temperatures, and precipitation across the United States. *Proc. Nat. Acad. Sci.* **106**, 7324-7329 (2009).

5. Lean, J. L. & Rind, D. H. How natural and anthropogenic influences alter global and regional surface temperatures: 1889 to 2006. *Geophys. Res. Lett.* **35**, L18701 (2008).

6. Ropelewski, C. F. & Halpert, M. S. Global and regional scale precipitation patterns associated with the El Niño/Southern Oscillation. *Mon Wea. Rev.* **115**, 1606–1626 (1987).

7. Johnson, N. C., Feldstein, S. B. & Tremblay, B. The continuum of Northern Hemisphere teleconnection patterns and a description of the NAO shift with the use of self-organizing maps. *J. Clim.* **21**, 6354-6371 (2008).

8. Dai, P. & Tan, B. The nature of the Arctic Oscillation and diversity of the extreme surface weather anomalies it generates. *J. Clim*. doi:10.1175/JCLI-D-16-0467.1 (2017).

9. Trenberth, K. E., Fasullo, J . T., Branstator, G. & Phillips, A. S. Seasonal aspects of the recent pause in surface warming. *Nature Clim. Change*, **4**, 911-916 (2014).

10. Trenberth, K. E. & Shea, D. J. Atlantic hurricanes and natural variability in 2005. *Geophys. Res. Lett.* **33**, L12704 (2006).

11. Van Oldenborgh, G. J., Te Raa, L. A., Dijkstra, H. A. & Philip, S. Y. Frequency- or amplitude-dependent effects of the Atlantic meridional overturning on the tropical Pacific Ocean. *Ocean Sci.* **5**, 293-301 (2009).

12. McKinnon, K. A., Rhines, A., Tingley, M. P. & Huybers, P. Long-lead predictions of eastern United States hot days from Pacific sea surface temperatures. *Nature Geosci.* **9**, 389-394 (2016).

13. Honda, M., Inoue, J. & Yamane, S. Influence of low Arctic sea-ice minima on anomalously cold Eurasian winters. *Geophys. Res. Lett.* **36**, L08707 (2009).

14. Cohen, J. *et al.* Recent Arctic amplification and extreme mid-latitude weather. *Nature Geosci.* **7**, 627-637 (2014).

15. Kug, J.-S., *et al.* Two distinct influences of Arctic warming on cold winters over North America and East Asia. *Nature Geosci.* **8**, 759-763 (2015).

16. Petoukhov, V. & Semenov, V. A. A link between reduced Barents-Kara sea ice and cold winter extremes over northern continents. *J. Geophys. Res.* **115**, D21111 (2010).

17. Kim, B.-M., *et al.* Weakening of the stratospheric polar vortex by Arctic sea-ice loss. *Nature. Comm.* **5**, 4646 (2014).

18. Mori, M., Watanabe, M., Shiogama, H., Inoue, J. & Kimoto, M. Robust Arctic sea-ice influence on the frequent Eurasian cold winters in past decades. *Nature Geosci.* **7**, 869-873 (2014).

19. Alexander, M. A. et al. The atmospheric response to realistic Arctic sea ice anomalies in an AGCM during winter. *J. Clim.* **17**, 890-905 (2004).

20. Magnusdottir, G., Deser, C. & Saravanan R. The effects of North Atlantic SST and sea ice anomalies on the winter circulation in CCM3. Part I: Main features and storm track characteristics of the response. *J. Clim*. **17**, 857-876 (2004).

21. Jaiser, R., Dethloff, K. & Handorf, D. Stratospheric response to Arctic sea ice retreat and associated planetary wave propagation changes. *Tellus* **65**, 19375 (2013).

22. Feldstein, S. B. & Lee, S. Intraseasonal and interdecadal jet shifts in the Northern Hemisphere: The role of warm pool tropical convection and sea ice. *J. Clim.* **27**, 6497-6518 (2014).

23. Inoue, J., Hori, M. E. & Takaya, K. The role of Barents Sea ice in the wintertime cyclone track and emergence of a warm-Arctic cold-Siberian anomaly. *J. Clim*. **25**, 2561-2568 (2012).

24. Cohen, J., Jones, J., Furtado, J. C. & Tziperman, E. Warm Arctic, cold continents: A common pattern related to Arctic sea ice melt, snow advance, and extreme winter weather. *Oceanography* **26**, 151-160 (2013).

25. Barnes, E. A. & Screen, J. A. The impact of Arctic warming on the midlatitude jet-stream: Can it? Has it? Will it? *WIREs Clim. Change* **6**, 277-286 (2015).

26. Sun, L., Perlwitz, J. & Hoerling, M. What caused the recent "Warm Arctic, Cold Continents" trend pattern in winter temperatures? *Geophys. Res. Lett.* **43**, 5345-5352 (2016).

27. Li, C., Stevens, B. & Marotzke, J. Eurasian winter cooling in the warming hiatus of 1998-2012. *Geophys. Res. Lett.* **42**, 8131-8139 (2015).

28. McCusker, K. E., Fyfe J. C. & Sigmond, M. Twenty-five winters of unexpected Eurasian cooling unlikely due to Arctic sea-ice loss. *Nature Geosci.* **9**, 838-842 (2016).

29. Screen, J. A., Simmonds, I., Deser, C. & Tomas, R. The atmospheric response to three decades of observed Arctic sea ice loss. *J. Clim.* **26**, 1230-1248 (2013).

30. Perlwitz, J., Hoerling, M. & Dole, R. Arctic tropospheric warming: Causes and linkages to lower latitudes. *J. Clim*. **28**, 2154-2167 (2015).

31. Brönnimann, S. Impact of El Niño-Southern Oscillation on European climate. *Rev. Geophys.*, **45**, RG3003 (2007).

32. Seneviratne, S. I., Donat, M. G., Mueller, B. & Alexander, L. V. No pause in the increase of hot temperature extremes. *Nature Clim. Change* **4**, 161-163 (2014).

33. Vogel, M. M, Orth R., Cheruy, F., Hagemann, S., Lorenz, R., Van den Hurk, B. J. J. M. & Seneviratne, S. I. Regional amplification of projected changes in extreme temperatures strongly controlled by soil moisture-temperature feedbacks. *Geophys. Res. Lett.* **44**, 1511-1519 (2017).

34. Donat, M. G., Pitman, A. J. & Seneviratne, S. I. Regional warming of hot extremes accelerated by surface energy fluxes. *Geophys. Res. Lett.* **44**, 7011-7019 (2017).
